# Ethical perspective on AI hazards to humans: A review

**DOI:** 10.1097/MD.0000000000036163

**Published:** 2023-12-01

**Authors:** Changye Chen, Ziyu Chen, Wenyu Luo, Ying Xu, Sixia Yang, Guozhao Yang, Xuhong Chen, Xiaoxia Chi, Ni Xie, Zhuoying Zeng

**Affiliations:** a The First Affiliated Hospital of Shenzhen University, Shenzhen University, Shenzhen, Guangdong, China; b The School of Public Health, Guilin Medical University, Gui Lin, Guangxi, China.

**Keywords:** artificial intelligence, ethics, potential hazards, regulation and supervision, sustainable development

## Abstract

This article explores the potential ethical hazards of artificial intelligence (AI) on society from an ethical perspective. We introduce the development and application of AI, emphasizing its potential benefits and possible negative impacts. We particularly examine the application of AI in the medical field and related ethical and legal issues, and analyze potential hazards that may exist in other areas of application, such as autonomous driving, finance, and security. Finally, we offer recommendations to help policymakers, technology companies, and society as a whole address the potential hazards of AI. These recommendations include strengthening regulation and supervision of AI, increasing public understanding and awareness of AI, and actively exploring how to use the advantages of AI to achieve a more just, equal, and sustainable social development. Only by actively exploring the advantages of AI while avoiding its negative impacts can we better respond to future challenges.

## 1. Introduction

With the rapid development of computer technology and data processing capabilities, artificial intelligence (AI) has become a highly anticipated field.^[1,2]^ AI is a type of computer system that simulates human intelligence and is used to achieve various tasks by mimicking human cognition, learning, and decision-making processes.^[3]^ It encompasses various technologies and algorithms, including machine learning, deep learning, natural language processing, computer vision, and more, which continuously challenge people understanding and expectations of machine capabilities. While we have witnessed the tremendous impact of AI on many fields, including medicine, transportation, manufacturing, and finance,^[4]^ there also exist potential hazards and risks associated with these developments.^[5,6]^ In this paper, we will focus on exploring the potential hazards of AI in medicine, intelligent transportation systems (ITS), smart manufacturing, logistics, and finance.

Firstly, let us take a closer look at the potential hazards of artificial intelligence (AI) in the medical field. Although there has been significant progress in the application of AI in medical diagnosis and treatment, it also brings about many risks and challenges.^[7]^ For example, in using AI algorithms for medical image diagnosis, there is a risk of misdiagnosis and missed diagnosis. This is because AI algorithms may be limited by the dataset and imperfect training models, which can lead to inaccurate identifications of diseases or lesions. In addition, AI technology may also pose data security problems, such as medical data leakage and abuse, which pose a threat to patients’ privacy and safety.^[8]^

Secondly, the potential hazards of AI in intelligent transportation, smart manufacturing, logistics, and other fields also need attention.^[8,9]^ In the field of intelligent transportation, AI technology can be used for traffic flow prediction, autonomous driving, and traffic management, but there are also some potential risks, such as vehicle hacking leading to accidents or traffic congestion. In the field of smart manufacturing, AI can help companies achieve automation and intelligence, improve production efficiency, and reduce costs, but it may also lead to employment competition between industrial robots and human workers, causing labor market instability.^[10]^ In the field of logistics, AI technology can improve logistics efficiency and accuracy, but there are also risks such as data privacy leakage and logistic security issues.

Lastly, let us examine the potential hazards of artificial intelligence (AI) in the finance sector. AI technology has been widely applied in the finance industry, including risk assessment, fraud detection, and investment decisions.^[11]^ Although AI can provide efficient and accurate solutions in these areas, there are also potential hazards and risks. For example, in risk assessment and fraud detection, the bias of the dataset may affect the accuracy of AI, leading to the risk of misjudgment and omission. Moreover, the opacity and unexplainability of AI algorithms may result in questions and disputes over financial decisions.^[12]^ For instance, if someone is denied a loan or credit card application without knowing the reason, they may question and challenge the decision. Additionally, AI technology may also be used for illegal activities such as financial fraud and money laundering, leading to instability and safety risks in financial markets.^[13]^

In summary, although AI has brought many benefits in various fields, it also carries potential hazards and risks. When applying AI technology, we must fully consider these risks and challenges and take appropriate measures to mitigate their impact. This may include strengthening data privacy protection, developing stricter regulations and standards, and increasing the transparency and explainability of algorithms.^[14,15]^ Only by effectively managing and controlling these risks can we fully leverage the advantages of AI technology, achieve more value, and drive innovation.

## 2 . The potential hazards of artificial intelligence in the medical field

The development of artificial intelligence (AI) has profoundly transformed modern medicine, bringing many potential benefits to healthcare such as improving the accuracy and efficiency of diagnosis and treatment, enhancing the reliability and scientificity of clinical decisions, and so on.^[16,17]^ However, at the same time, AI in the medical field has also brought many potential hazards involving ethics, law, society, politics, and other aspects. This article will focus on discussing the potential hazards of AI in the medical field and exploring how to manage and address them from the perspective of medical ethics.

### 2.1. Medical ethics and privacy issues

The widespread application of artificial intelligence (AI) in the medical field is dependent on the dramatic increase in medical data, which often contains personal privacy information regarding individuals’ physical, psychological, and behavioral aspects.^[18]^ With technological advancements, AI can better process this data and extract useful information for clinical decision-making, personalized treatment, drug development, and more. However, this data may also be misused or leaked, potentially resulting in violations of patient privacy.^[19]^ Additionally, the complexity and opacity of AI algorithms may make it difficult to explain, rendering the use and analysis of personal data more opaque and challenging. These issues bring about serious ethical and privacy concerns that need to be managed and addressed from the perspective of medical ethics.

### 2.2. Autonomy and responsibility issues

The widespread application of AI in the medical field also brings about autonomy and responsibility issues. In some cases, AI systems can make autonomous decisions, for instance, in diagnosis and treatment, AI can automatically recommend the best treatment plan without the intervention of a doctor.^[20]^ Such autonomous decision-making may result in the neglect of patients’ interests, as AI systems may not understand patients’ specific circumstances and needs.^[21]^ Furthermore, the extensive use of AI in the medical field may also lead to responsibility issues, as in some cases, it may be difficult to determine the boundary of responsibility between AI systems and doctors. These issues need to be managed and addressed from the perspective of medical ethics.

### 2.3. Social justice issues

In addition to safety concerns, the application of artificial intelligence (AI) in the medical field may also give rise to social justice issues. In medical decision-making, AI algorithms may tend to make more proactive diagnostic and treatment decisions for certain groups of people, while adopting a more conservative approach for others.^[22]^ For instance, in some groups, AI-based algorithms may overestimate the risk of disease, leading to more tests and treatments for these individuals, while underestimating the risk of disease for other groups, resulting in delayed diagnosis and treatment. Such biases may impact healthcare outcomes for certain groups of people, leading to unfair treatment in the medical field. Furthermore, this unfair treatment may be exacerbated if the use of AI algorithms is misguided or abused, potentially exacerbating social division and inequality.^[23]^ For example, studies suggest that machine learning-based algorithms may be biased in diagnosis and treatment for certain groups of people based on factors such as race, gender, age, and income. These factors may be irrelevant to disease risk, but machine learning algorithms may erroneously associate them with disease risk, leading to unfair treatment for certain groups of people. For instance, in the prediction of heart disease, the use of AI algorithms may overestimate the risk of disease for Black and Hispanic populations, while underestimating the risk for Asian populations, resulting in unfair treatment for these groups in the medical field. Another issue related to social justice is that if medical institutions and doctors excessively rely on AI algorithms, they may lose their ability to think independently and make independent judgments, which may compromise the quality and reliability of healthcare. Doctors should remain vigilant when using AI algorithms and verify and review algorithm results when necessary.

In summary, while AI holds tremendous potential in the medical field, it may also bring about potential dangers and challenges. In order to realize the full potential of AI in the medical field, extensive research and analysis of its potential dangers and challenges are necessary, and corresponding regulatory and normative measures need to be developed to ensure the safety and reliability of AI in the medical field, while safeguarding the rights and interests of the public.

## 3. Potential hazards of artificial intelligence in the areas of intelligent transportation, manufacturing, and logistics

### 3.1. Potential hazards of artificial intelligence in the field of intelligent transportation

With the continual development of artificial intelligence (AI), ITS have become an area of significant concern. ITS leverages technologies such as sensors, data analytics, and machine learning to optimize traffic flow and reduce the risk of traffic incidents. However, the use of AI in ITS also poses some potential hazards.

Firstly, the application of AI in ITS may bring about privacy and security risks.^[24]^ ITS requires the collection of significant amounts of personal and vehicle data, such as vehicle location, speed, and driving behavior, to optimize and manage traffic flow. However, the collection and use of this data may infringe on the privacy rights of vehicle owners and lead to security risks, such as data leaks and malicious attacks.

Secondly, the use of AI in ITS may lead to a reduction in employment opportunities. As automated driving technology continues to advance, ITS may replace some drivers’ jobs, resulting in job losses.^[25]^ This could cause economic difficulties for those who depend on driving jobs for a living.

Furthermore, the application of AI in ITS may also raise some ethical and legal issues.^[26]^ For example, when an autonomous vehicle is involved in an accident, how should responsibility be determined? Should the car manufacturer, software developer, or car owner be held responsible? These issues require further research and discussion to ensure that the application of AI in ITS can be carried out within ethical and legal frameworks.

In summary, while the application of AI in ITS may bring some benefits, such as reducing traffic accidents and alleviating traffic congestion, it may also pose some potential hazards. To ensure the safe and reliable implementation of AI in ITS, it is necessary to conduct in-depth research and evaluation of its potential hazards and develop corresponding legal and ethical frameworks to ensure fairness and safety.

### 3.2. Potential hazards of artificial intelligence in intelligent manufacturing

Intelligent manufacturing refers to the application of emerging technologies such as artificial intelligence, the Internet of Things, and big data in the manufacturing industry to achieve automation, intelligence, networking, and efficiency throughout the production process, thereby improving production efficiency and product quality. Although intelligent manufacturing technology has brought many benefits, it also brings some potential hazards.

Firstly, the application of artificial intelligence in intelligent manufacturing may lead to the loss of control or malfunction of robots or equipment on the production line.^[27]^ For example, if a robot malfunctions, it may cause the production line to stop, which further results in a reduction in production volume and work efficiency. In addition, when intelligent devices cannot cooperate effectively, it may cause chaos or safety hazards on the production line.

Secondly, the application of artificial intelligence in intelligent manufacturing may also result in some labor being replaced or unemployed.^[28]^ Repetitive and monotonous work may be replaced by robots or intelligent equipment, such as assembly, inspection, and handling. This may cause some workers to face the risk of unemployment, affecting social stability and fairness.

Moreover, data security issues in intelligent manufacturing also pose potential hazards.^[29]^ In intelligent manufacturing, a large amount of data is collected and stored, such as production data, worker data, and equipment data. If these data are attacked or leaked by hackers, it may cause serious impacts on a company business secrets and customer privacy, even leading to financial loss.

Finally, the intelligence and automation in intelligent manufacturing may lead to a reduced understanding of the manufacturing process and product quality by humans.^[30]^ With the continuous optimization and development of smart devices and algorithms, human intervention and supervision may decrease, making it more difficult to discover and solve product quality issues. This may affect a company reputation and consumer safety.

Therefore, in the field of intelligent manufacturing, we need to control the application of artificial intelligence, strengthen data security protection, establish a human-machine collaboration manufacturing model to ensure human safety and health, and actively promote the integration of intelligent manufacturing and social justice.

### 3.3. Potential hazards of artificial intelligence in intelligent logistics

Intelligent logistics refers to a mode of using artificial intelligence (AI) technology to realize automation and intelligence in the logistics process. AI technology is widely applied in logistics management, intelligent scheduling, and automated warehousing, bringing significant benefits to the logistics industry such as improved efficiency and accuracy, as well as reduced costs. However, AI technology in intelligent logistics also brings potential hazards.

Firstly, AI algorithms used in intelligent logistics systems may have errors or biases.^[31]^ For example, in machine learning-based route planning systems, problems with data sources or algorithms can result in incorrect route planning or deviations from the original plan, affecting the accuracy and timeliness of logistics. In addition, if the robot control algorithm in automated warehousing systems is flawed, it may result in robots misjudging the location of goods or operating improperly, leading to damage to goods or abnormal storage operations, resulting in losses for logistics operations.

Secondly, the robots or automated equipment used in intelligent logistics systems may have the risk of malfunction or loss of control.^[32]^ Although automated logistics equipment is often equipped with multiple safety measures, such as sensor detection and safety rollbacks, flaws in the system or inappropriately implemented measures may cause robots to malfunction or lose control, posing safety hazards to operations and employees, and even causing personal injury.

Furthermore, collaboration issues may arise between man and machine in intelligent logistics systems. Coordination between robots and employees is often necessary, such as in coordinating loading and unloading. However, if the decisions made by robots are in conflict with those made by employees, it may cause interruptions or confusion in the logistics process, affecting normal logistics operations.

Finally, big data and privacy protection are also important issues in intelligent logistics systems. Intelligent logistics systems typically generate large amounts of data, such as cargo information, logistics operations data, and employee data. If this data is not protected, it may be leaked to unauthorized third parties, resulting in personal privacy breaches or commercial secrets being disclosed. In conclusion, although AI technology brings many advantages to the intelligent logistics field, it also poses potential hazards.

## 4. Potential hazards of artificial intelligence in finance

With the continuous development of artificial intelligence (AI) technology, the financial industry has also begun to accelerate its application of AI technology. In the banking, insurance, and stock markets, AI technology has been widely used to improve the efficiency and quality of financial services through techniques such as data mining, machine learning, and deep learning. However, the widespread application of AI technology in the financial industry also poses potential hazards that may have negative impacts on the stability and fairness of financial markets.

### 4.1. Potential hazards of artificial intelligence in financial fraud

With the rapid development of financial technology, the application of artificial intelligence (AI) in the financial industry is becoming increasingly widespread, including in the areas of risk management, investment decision-making, credit assessment, and anti-fraud.^[33]^ Among these applications, anti-fraud is a field where AI technology is widely used. However, the use of AI technology in the anti-fraud domain also brings potential hazards.

Firstly, AI technology may cause false positives, i.e., erroneously detecting normal transactions as fraudulent activities.^[34]^ For example, when consumers use credit cards to shop overseas, machine learning algorithms may classify the transaction as fraudulent, leading to rejected transactions or additional scrutiny. This will not only affect consumers’ shopping experience but may also damage their credit, affecting future credit applications.

Secondly, AI technology may cause false negatives, i.e., missing fraudulent activities. This is because fraudulent activities often use covert and unpredictable methods, and AI technology may have vulnerabilities in identifying fraudulent behavior, resulting in undiscovered fraud. This can cause financial institutions to face significant risks and losses.^[35]^

Thirdly, attackers may exploit AI technology to carry out fraudulent activities.^[36]^ For example, attackers can take advantage of vulnerabilities in AI technology, forge identity information, deceive algorithms, or disrupt identification models to commit fraud. This will cause significant harm to financial institutions and consumers.

Finally, AI technology may pose a threat to personal privacy and data security. In anti-fraud applications, machine learning algorithms typically require the use of a large amount of personal data, including transaction records, credit scores, and social network information, to train models and identify fraudulent behavior. However, the collection, storage, and processing of this data may lead to risks of privacy breach and data leakage. To address these potential hazards, financial institutions need to take effective measures to safeguard customers’ rights and data.

### 4.2. Potential hazards of artificial intelligence in financial instability

With the continuous development of artificial intelligence technology, its application in the financial field is becoming increasingly wide-ranging. Artificial intelligence can help financial institutions to better carry out data analysis, risk management, and decision-making, thus improving the efficiency and accuracy of the finance industry. However, the application of artificial intelligence in financial instability also has some potential hazards. This article will elaborate on 4 aspects in detail:

1. Expansion of financial risk: The application of AI models in the financial sector requires a large amount of data, which may be affected by factors such as market fluctuations and policy changes.^[37]^ If the training data of the model is insufficient or not representative, it will lead to inaccurate prediction results of the model, which will further expand financial risk. For example, in the 2008 financial crisis, some financial institutions’ risk models did not consider the real estate market bubble, resulting in the collapse of the financial market. If these institutions use AI models, due to their stronger dependence on data, they may further expand financial risk. In addition, the application of AI models may also lead to the ”black box” effect, that is, the predicted results of the model cannot be explained and understood. In this case, financial institutions cannot understand how the model makes decisions, nor can they discover potential risks and loopholes. If there is a problem with the model, financial institutions may find it difficult to trace the root cause of the problem due to the inability to explain the model decision, thereby expanding financial risk.
2. Increase in financial market instability: The application of AI models may lead to an increase in financial market instability.^[38]^ For example, some institutions may use similar algorithms and models, which can lead to large-scale market fluctuations and instability. In addition, the application of artificial intelligence models can also increase market opacity, making it difficult for investors to understand the true situation of the market, thereby increasing market instability. The application of artificial intelligence models may also lead to self-reinforcing market trends, in which some institutions or investors in the market take corresponding investment actions based on the predicted results of the models, thereby affecting the market direction. If multiple institutions or investors in the market use similar models, a ”herd effect” may occur, leading to increased market volatility and instability.
3. Increased technical risks: The application of AI models requires a lot of technical support and data storage. If there is a technical failure or a hacking attack, it can lead to the collapse of the financial system and the risk of data leakage.^[39,40]^ For example, in 2017, the US credit rating agency Equifax was hacked, resulting in the leakage of 143 million personal information, which could more severely affect financial institutions and individual privacy. Since the application of AI models requires a large amount of data storage and computing resources, financial institutions need to invest a lot of capital to support their construction and maintenance. This may also lead to differences in technological investment among some financial institutions, exacerbating market competition and instability.
4. Increased ethical risks: The application of AI models requires a lot of data support. If these data sources are improper or have ethical issues, it will damage the reputation of financial institutions and may even cause public outrage.^[41,42]^ For example, in 2018, Google cooperation with the US Department of Defense raised ethical issues and received widespread social attention and resistance. If the data used by financial institutions has similar issues, they may face similar ethical risks. In addition, the application of AI models may also lead to neglect of human factors, that is, the model relies too much on data and algorithms, and neglects subjective factors and judgments of humans. In this case, the model may ignore some important factors and risks, leading to biased decision-making of financial institutions and further expanding financial risks.

In summary, although the application of artificial intelligence (AI) in the financial instability domain brings many conveniences and advantages, it also entails certain hazards and risks, which require close attention and management from financial institutions and regulatory authorities. Financial institutions need to strictly monitor and manage the development, application, and maintenance of AI models to ensure their reliability and safety. Regulatory authorities must strengthen their supervision and monitoring of financial institutions to ensure the stability and healthy development of the financial market. Additionally, financial institutions need to strengthen their research and development efforts on AI technology to explore how to better manage and control financial risks using such technology. For instance, research can explore how to use AI to predict and monitor market fluctuations and risks, as well as how to optimize financial institutions’ risk management and investment decisions. In conclusion, the application of AI in the financial instability domain has both opportunities and challenges, requiring joint efforts from financial institutions and regulatory authorities to ensure its sustainability and stability and to promote the stability and healthy development of the financial market.

### 4.3. Potential hazards of artificial intelligence in financial data privacy

The increasing applications of artificial intelligence (AI) technology in the financial sector, such as risk control, investment decision-making, and customer service, bring potential privacy and security risks, particularly in the realm of financial data privacy. This article discusses the potential hazards of AI in financial data privacy from several perspectives:

1. Sensitive data leakage: customer data of financial institutions involves a great deal of sensitive information, such as personal identity information, financial status, and transaction records.^[43,44]^ In the process of AI technology application, customer data could potentially be leaked, leading to personal privacy rights violations. For example, a bank may provide customer data to third-party institutions for data analysis and mining, but these institutions may use the data for other purposes or suffer data leakage during data transmission due to hacker attacks.
2. Algorithmic discrimination and unfairness: AI algorithmic training and optimization process may result in discrimination and unfairness issues.^[45]^ For instance, in credit assessment, AI algorithms may tend to assign higher credit scores to certain groups of people, resulting in discrimination against others. This may cause some people to be denied loans or charged higher interest rates, leading to unfair treatment.
3. Data tampering and manipulation: AI technology applications could result in data tampering and manipulation.^[46,47]^ For example, in stock trading, AI algorithms might fall prey to hacking or malicious programs, leading to unusual fluctuations in stock prices. This could cause huge losses to investors, and even cause market crashes.
4. Inadequate privacy protection measures: Financial institutions using AI technology may not have adequate privacy protection measures in place.^[48,49]^ For instance, financial institutions may not have fully anonymized and encrypted customer data, leading to increased data leakage risks. Additionally, financial institutions may not carry out sufficient auditing and supervision of AI algorithms, leading to loopholes and security risks.
5. Social engineering attacks: Social engineering attacks involve obtaining sensitive information through deception and trickery.^[50]^ In the financial field, social engineering attacks may lead to customer data leaks and financial fraud. For instance, hackers may try to obtain customer account information and passwords by sending phishing emails or impersonating financial institution staff.

In summary, the potential hazards of AI in financial data privacy include sensitive data leakage, algorithmic discrimination and unfairness, data tampering and manipulation, inadequate privacy protection measures, and social engineering attacks. To mitigate these risks, financial institutions need to strengthen their supervision and auditing of AI technology, enhance customer data protection, and improve employee security awareness through training. Additionally, governments and regulatory bodies need to establish relevant policies and regulations to safeguard citizens’ privacy rights and interests.

## 5. Conclusion

With the rapid development of artificial intelligence (AI) technology, we have seen its applications and impacts in many fields. However, this technology also brings many risks and challenges, especially in the fields of medicine, intelligent transportation, intelligent manufacturing, intelligent logistics, and finance. We need to think deeply and discuss how to manage and supervise the development of AI technology to ensure the maximum protection of the interests of human society.

Firstly, we need to recognize that the application and development of AI technology are not isolated events but are closely related to ethical and moral values. Therefore, we need to establish a comprehensive ethical framework to guide and restrict the development and application of AI technology. These ethical frameworks should include protecting data privacy and information security, avoiding unfairness and discrimination, respecting human dignity and values, and ensuring public safety and health.

Secondly, we need to strengthen the supervision and management of AI technology. Governments, academia, industry, and the public should all participate in this process to ensure that the development and application of AI technology meet ethical and moral requirements. Governments should establish relevant laws, regulations, and policies to regulate and guide the application of AI technology. Academia should strengthen research and evaluation of AI technology to ensure its scientificity and reliability. Industry should establish industry standards and norms to ensure the safety and quality of AI technology. The public should participate in the development and application of AI technology to express their opinions and views.

When considering the ethics of AI application, it is important to weigh the potential risks against benefits. Not utilizing AI may also miss opportunities to improve social efficiency, fairness and sustainable development. We recommend taking an approach to actively explore the advantages of AI, while avoiding its negative impacts, in order to leverage AI to achieve a more just, equal and sustainable social development. Specifically, a utilitarian calculation weighing benefits versus harms could help determine whether AI application is ethical in a given context. We should also consider deontological principles, including respect for human dignity and autonomy, to guide the development and use of AI technology. Overall, a nuanced ethical analysis, rather than a one-sided focus on harms, will allow us to fully realize the potential of AI while proactively addressing its risks.

Finally, we need to realize that the development and application of AI technology are long term processes, and we need to constantly learn and adapt to new technologies and application scenarios. We need to establish a scientific, standardized, and responsible ecosystem for the development and application of AI technology to ensure sustainable development in economic, social, and environmental aspects. We need to encourage innovation and practice while also paying attention to risks and challenges to timely respond and solve problems. We also need to strengthen international cooperation and communication to jointly address global AI challenges and risks.

In the medical field, AI technology can be used for disease diagnosis and treatment plan formulation, but at the same time, we need to consider privacy protection and patient rights. In the field of intelligent transportation, AI technology can improve traffic efficiency and safety, but we also need to pay attention to data security and ethical risks. In the field of intelligent manufacturing, AI technology can improve production efficiency and product quality, but we also need to pay attention to workers’ rights and environmental pollution. In the fields of intelligent logistics and finance, AI technology can improve logistics efficiency and financial service quality, but we also need to avoid unfairness and discrimination.

In conclusion, managing and supervising the development and application of AI technology is a complex and important task. We need to establish a comprehensive ethical framework, strengthen supervision and management of AI technology, constantly learn and adapt to new technologies and application scenarios, and establish a scientific, standardized, and responsible ecosystem for the development and application of AI technology. Only in this way can we ensure that AI technology is truly used for the benefit of human society.

## Author contributions

**Conceptualization:** Zhuoying Zeng.

**Formal analysis:** Ziyu Chen, Wenyu Luo, Ying Xu, Sixia Yang, Guozhao Yang, Xuhong Chen, Xiaoxia Chi, Zhuoying Zeng.

**Funding acquisition:** Zhuoying Zeng.

**Project administration:** Zhuoying Zeng.

**Writing – original draft:** Changye Chen.

**Writing – review & editing:** Ziyu Chen, Wenyu Luo, Ying Xu, Sixia Yang, Guozhao Yang, Xuhong Chen, Xiaoxia Chi, Ni Xie, Zhuoying Zeng.
